# Reduced Graphene Oxide-Based Foam as an Endocrine Disruptor Adsorbent in Aqueous Solutions

**DOI:** 10.3390/membranes10110340

**Published:** 2020-11-13

**Authors:** Jeanne N’Diaye, Sujittra Poorahong, Ons Hmam, Gastón Contreras Jiménez, Ricardo Izquierdo, Mohamed Siaj

**Affiliations:** 1 Department of Chemistry and Biochemistry, Université du Québec à Montréal, Montréal, QC H3C 3P8, Canada; ndiaye.jeanne93@gmail.com (J.N.); sujittra.po@mail.wu.ac.th (S.P.); hmamons@yahoo.fr (O.H.); co.gaston@ecologia.unam.mx (G.C.J.); 2 Laboratorio de Microdisección Láser, Instituto de Ecología, Universidad Nacional Autónoma de Mexico, Ciudad de Mexico 04510, Mexico; 3 École de technologie supérieure, Université du Québec à Montréal, Montréal, QC H3C 1K3, Canada; ricardo.izquierdo@etsmtl.ca

**Keywords:** reduced graphene oxide, membrane, endocrine disruptors, adsorption, Langmuir, Freundlich

## Abstract

A stable and magnetic graphene oxide (GO) foam–polyethyleneimine–iron nanoparticle (GO–PEI–FeNPs) composite has been fabricated for removal of endocrine disruptors—bisphenol A, progesterone and norethisterone—from aqueous solution. The foam with porous and hierarchical structures was synthesized by reduction of graphene oxide layers coupled with co-precipitation of iron under a hydrothermal system using polyethyleneimine as a cross linker. The presence of magnetic iron nanoparticles facilitates the separation process after decontamination. The foam was fully characterized by surface and structural scanning electron microscopy, Fourier transform infrared spectroscopy, Raman spectroscopy and X-ray photoelectron spectroscopy. The foam exhibits a high adsorption capacity, and the maximum adsorption percentages are 68%, 49% and 80% for bisphenol A, progesterone and norethisterone, respectively. The adsorption process of bisphenol A is explained according to the Langmuir model, whereas the Freundlich model was used for progesterone and norethisterone adsorption.

## 1. Introduction

Endocrine disruptors are substances affecting the primary communication network of the body, which is responsible for controlling multiple body functions. This network is named the endocrine system and includes different organs such as ovaries, testes, adrenal glands, pituitary gland, thyroid, and the pancreas. These chemicals harm human and wildlife by causing tumors, birth defects and disorders in development of the fetus. In addition to these effects on the human body, these disruptors can also mimic or interfere with the function of hormones in the body due to similarity in their structure and activity by influencing sexual development of humans or wildlife. The best example is feminization of males or masculinization effects on females [1,2,3]. One of the major endocrine disruptors, called bisphenol A (BPA), possesses hormone-like behavior and is contained in epoxy resins that are used to line water pipes. Furthermore, natural hormones such as progesterone, β-Estradiol and norethisterone when in excess can also act on the endocrine system, thus causing a disorder in the regular hormonal process of living beings.

Various techniques have been developed throughout the years to adsorb and remove BPA and other endocrine disruptors from water. Yuan and Al used composite electrodes such as Pd/Ti and RuO_2_/Ti to degrade BPA in a soil matrix [4,5]. Another approach is to utilize carbon materials such as activated carbon and carbon nanotubes to remove BPA. Although these materials have been found to be very effective at absorbing BPA, with an adsorption capacity reported to be 182 mg g^−1^, an issue remains in terms of recyclability [6,7,8].

Graphene is one of the most studied carbon nanomaterials, discovered in 2004 by Novoselov and Geim [9]. This two-dimensional carbon material with a honeycomb lattice structure exhibits tremendous properties including a high surface area of 2630 m^2^.g^−1^, outstanding mechanical properties with a high Young’s modulus (1 TPa), a high thermal conductivity (5000 W mK^−1^) and an electron mobility higher than those of most semi-conductor materials (2.5 × 10^5^ cm^2^ V^−1^ s^−1^) [10,11,12].

One method to produce high-quality graphene is chemical vapor deposition (CVD); however, this method only gives a few layers of graphene. Therefore, researchers have developed alternative synthesis methods for large-scale production of graphene via chemical exfoliation of graphite. Multiple synthesis paths have been developed since the 19th century, starting with Brodie who was able to oxidize graphite [13] and then Staudenmaier who improved the synthesis by reducing the number of oxidation steps [14]. The last enhancement of graphene oxide (GO) synthesis was developed by Hummers who used potassium permanganate, which is a strong oxidizing agent, and sulfuric acid instead of fume nitric acid, which was used previously [15]. Others have made modification to this synthesis, but the mechanism to form GO remains the same [16].

Many applications have been developed based on graphene material such as preparation of nanocomposites with polymers fillers, e.g., polyvinyl alcohol (PVA), poly(methyl methacrylate) (PMMA) or polyethyleneimine (PEI) [17,18,19]. These composite materials were synthesized in order to enhance mechanical, electrical or structural properties of the polymers. Graphene oxide can be used for preparation of thin conductive films by using various deposition techniques, [20,21,22,23,24] fabrication of electrochemical sensors and biosensors [25,26], preparation of 3D graphene oxide assembly for dyes and gas adsorption [27,28], and as an electrode for energy storage applications like supercapacitors [29,30]. Researchers have worked toward investigating and developing models for understanding the adsorption mechanism of large macromolecules. For instance, the Langmuir and Freundlich models have been used to investigate removal of dye using different substrates [31,32]. The Langmuir model often assumes adsorption of a monolayer on a homogenous surface, while the Freundlich isotherm is based on adsorption of multilayers on a heterogeneous surface [33].

In this paper, fabrication of a magnetic reduced graphene oxide 3D foam is presented. Graphene oxide was synthesized using a modified Hummers method. The composite material was fabricated using PEI as a polymer binder and iron nanoparticles under hydrothermal condition. The iron nanoparticles (FeNPs) were included in the composite material to introduce magnetic properties. After synthesis, the material was characterized by scanning electron microscopy, X-ray photoelectron spectroscopy (XPS), X-ray diffraction (XRD) and Raman spectroscopy. The foam is structurally stable and exhibits magnetic properties. The material was used to adsorb endocrine disruptors such as BPA, progesterone and norethisterone, and a comparison of adsorption behavior between the former molecules is presented. Evaluation of the adsorption ability of the GO composite foam toward endocrine disruptor compounds was carried out using UV–VIS spectroscopy. It is capable of attracting and adsorbing large molecules through π–π stacking, van der Waals forces or hydrogen bonding. This is the first attempt to adsorb high molecular weight molecules with a graphene-based membrane in an aqueous medium, showing that these types of material are good candidates for these types of application.

## 2. Materials and Methods

### 2.1. Materials

Natural flakes of graphite with an average particle diameter of 300 μm (99 wt% purity), sulfuric acid (98 wt%), hydrogen peroxide (30 wt%), phosphoric acid, potassium permanganate, hydrochloric acid (37 wt%), ethanol (100 wt%), ethyl ether, PEI (linear, Mw ≈ 50,000 g mol^−1^), iron nanoparticles (FeNPs) [34], bisphenol A, progesterone and norethisterone were all purchased from Sigma Aldrich (Oakville, ON, Canada). All chemicals were used without further purification. Ultrapure water (18 MΩ∙cm) was used in all experiments.

### 2.2. Graphene Oxide Synthesis

Graphene oxide was prepared using natural graphite powder through a modified Hummers method [16]. In a typical experiment, graphite (3 g, 500 mesh), 360 mL of H_2_SO_4_ and 40 mL of H_3_PO_4_ were put into a 1 L flask. The mixture was stirred for 30 min at 55 °C. After this step, 18 g of KMnO_4_ was added in small portions to prevent rapid temperature rise. The mixture was stirred continuously for 4.30 h and the temperature was kept at 55 °C. Several ultrasonication periods of 30 min were applied during the stirring time to exfoliate graphite oxide into graphene oxide sheets [21]. The suspension was further treated in order to convert the residual permanganate and MnO_2_ into soluble MnSO_4_ by adding it to a mixture of H_2_O_2_ (10 mL, 30%) and water (600 mL) at 0 °C and stirred. The resulting suspension had a bright yellow color. This suspension was further cleaned with water, and a mixture of water, 37% HCl and 100% ethanol. The mixture was then washed with ethyl ether and filtrated on a Teflon filter. The obtained GO is dried in an oven at 30 °C under vacuum. Then the GO was resuspended in water at a concentration of 1.76 mg mL^−1^ and the obtained yellow-brown aqueous suspension of GO was stored at room temperature for further use. 

### 2.3. 3D Porous Materials Preparation (GO–PEI–FeNPs)

Fabrication of 3D porous GO–PEI–FeNPs was adapted from Han and co-workers [17]. Next 5 mL of GO hydrogel was functionalized with 200 mg of PEI. After the solution was mixed by firmly shaking, aggregates were formed. Then 120 mg of iron nanoparticles powder was added to the mixture and sonicated in an ultrasound bath for 3 h. The solution was placed in an oil bath overnight at 90 °C. The obtained gel was centrifuged at 4000 rpm for 10 min. The hydrogel solution was transferred to a template, and the composite material was frozen at −80 °C for 4 h, and then freeze-dried under vacuum for 24 h.

### 2.4. Instrumental Characterization

The GO–PEI–FeNPs composite was imaged with a JEOL JSM7600F (Peabody, MA, USA) scanning electron microscope (SEM). Raman spectroscopy was conducted using a Renishaw InVia Raman microscope (Ulm, Germany) with a 488 nm laser to confirm the integrity of the GO structure. Attenuated total reflectance–Fourier transform infrared spectra (ATR–FTIR) were recorded with a Nicolet 6700 (Madison, WI, USA) to identify bonding between PEI and GO. X-ray diffraction (XRD) with a Bruker D8 Advance (D-5000) (Bruker, MA, USA) was performed to study the phase composition of the iron nanoparticles. The chemical composition of the sample was investigated by X-ray photoelectron spectroscopy using XPS (PHI 5600-ci, Physical Electronics, Eden Prairie, MN, USA). The adsorption behavior of endocrine disruptors from the GO–PEI–FeNPs composite was studied by measuring unabsorbed amounts of analyte in solution via UV–VIS with a LAMBDA 750 UV/Vis/NIR spectrometer (Perkin Elmer, Waltham, MA, USA).

### 2.5. Adsorption of BPA, Progesterone and Norethisterone

The adsorption capacity of the foam for BPA, progesterone and norethisterone was studied in aqueous solution. In a typical experiment, the GO–PEI–FeNPs foam was immersed in a beaker containing 100 mL of 15 ppm of each analyte solution: BPA, progesterone and norethisterone. Adsorption was carried out at room temperature. The solution was sampled at various adsorption times and the adsorption ability of the foam was quantified with UV–VIS spectroscopy. The effects of immersion time and concentration of the analyte were studied by calculating the percentage of compounds (%) adsorbed by the graphene-based membrane using Equation (1) for each GO–PEI–FeNPs foam immersion into a specific analyte solution at a determined time.

(1) $${\%\;\mathrm{adsorbed}\;\mathrm{compounds}}_{t}\;=\;\left(\frac{C_{i}-C_{f}}{C_{i}}\right)\times 100$$

where C_i_ and C_f_ (mg∙L^−1^) represent the initial and final concentrations, respectively, at a determined time, t, of BPA, progesterone and norethisterone aqueous solutions.

As the adsorption of analyte onto GO–PEI–FeNPs foam obeys a binding isotherm, the % removal for each set of incubations was plotted vs. the time (t) of each incubation. Later the rectangular hyperbola function form in Equation (2) was used to determine the maximum % adsorbed compounds (% R_max_) for each analyte through non-linear regression analysis. The *k*’ is a constant related to desorption [35,36,37].

Rectangular hyperbola function form:

(2) $$\%\;\mathrm{adsorbed}\;\mathrm{compounds}=\frac{{\%R}_{\max}\cdot t}{k^{\prime }+t}$$

## 3. Results and Discussion

### 3.1. Membrane Characterization 

**Figure 1:**
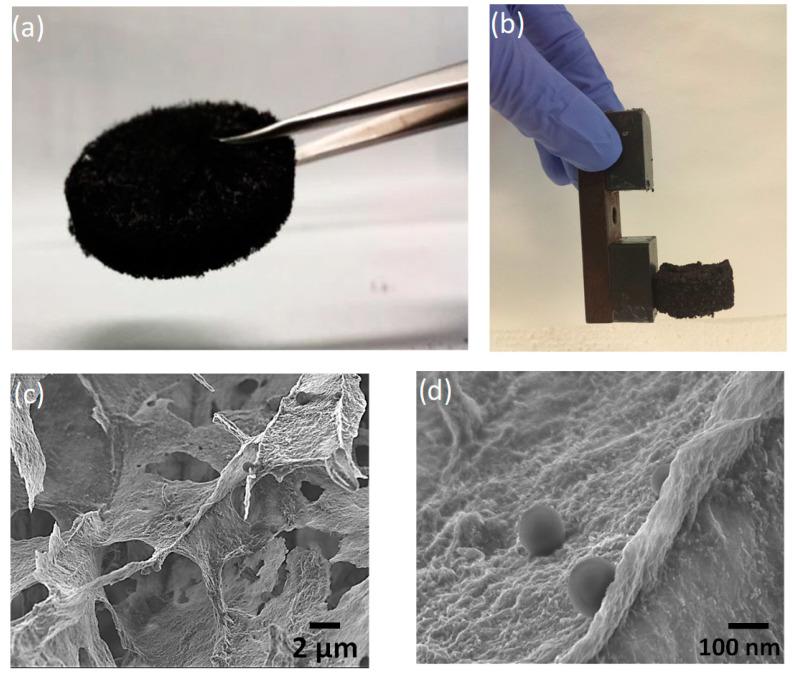
Digital images of the composite foam (**a**) and the foam in the contact with an external magnet (**b**). SEM images at different magnifications (**c**,**d**).

Figure 1 shows a digital image of the GO-based foam and SEM images at different magnifications. Each GO–PEI–FeNPs foam was stable and was ca. 0.4 cm thick with a diameter of ca. 3 cm (Figure 1a). In addition, the composite foam exhibited magnetic properties when in contact with an external magnet (Figure 1b). In the SEM image of the GO–PEI–FeNPs foam in Figure 1c, the synthesized GO-based composite foam material shows a macroporous structure with iron nanoparticles deposited on the surface. This porous structure provides a large surface area that will promote diffusion of the analyte from the bulk solution to the surface, which could lead to more efficient adsorption. At higher magnification, as seen in Figure 1d, the magnetic particles are observed with an average diameter of 100 nm.

**Figure 2:**
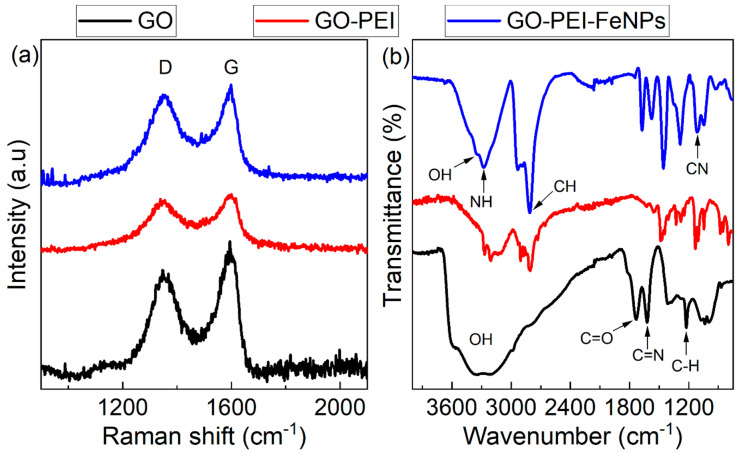
Raman spectra (**a**) and ATR–FTIR (**b**) of GO, GO–PEI and GO–PEI–iron nanoparticles (FeNPs).

Raman spectroscopy was conducted in order to evaluate the graphitic nature of the composite. Figure 2a shows the Raman spectra of three different types of foams, GO, GO–PEI and GO–PEI–FeNPs. All three spectra exhibit typical Raman D and G bands of GO at 1347.91 cm**^−^**^1^ and 1594.36 cm**^−^**^1^, respectively [38]. These results indicate that the method used to prepare the foam is non-destructive and the nature of the carbon is maintained throughout the process, even after incorporation of PEI and iron nanoparticles. Knowing that the intensities (I_D_/I_G_) ratio is proportional to the number of structural defects, this ratio was evaluated as 0.81, 0.97 and 0.98 for GO, GO–PEI and GO–PEI–FeNPs, respectively. The increase in I_D_/I_G_ ratio with addition of PEI and FeNPs into the foam matrix involves a reduction of the graphene oxide material. Reduction of the GO nanosheets in the foam was confirmed by comparing the ATR–FTIR spectra of GO, GO–PEI and GO–PEI–FeNPs (Figure 2b). ATR-FTIR data show a significant decrease in the intensity of the peak corresponding to the carbonyl and the hydroxyl functional groups at 1735 cm**^−^**^1^ and 3354 cm**^−^**^1^, respectively. They indicate the presence of a carbonyl functional group. This decrease is accompanied with increase of the C−N bands at 1124 cm**^−^**^1^, indicating that a C−N bond is formed between the polymer and the GO. In addition, the peaks corresponding to the PEI are also visible at 3293 and 2900 cm**^−^**^1^, demonstrating successful addition of the PEI in the foam. 

**Figure 3:**
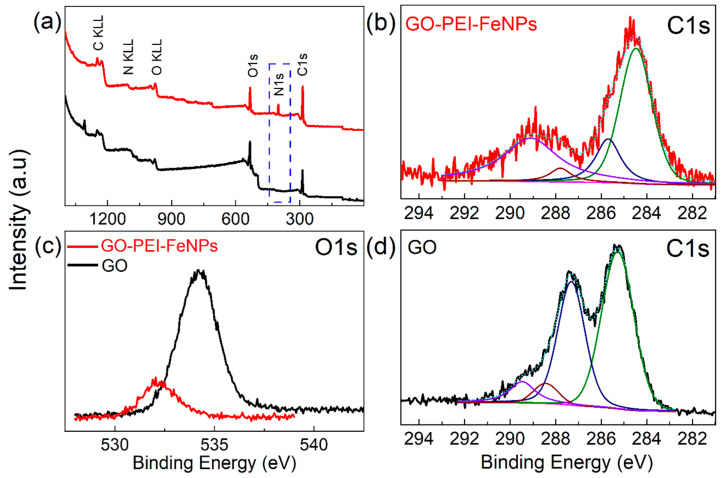
XPS of survey spectrums of the GO and GO–PEI–FeNPs foams (**a**). High-resolution spectrums of C1s (**b**,**d**) and O1s before and after functionalization with PEI (**c**).

The chemical compositions of GO and the prepared GO–PEI–FeNPs foam composite were further studied using XPS, a sensitive and powerful tool for monitoring structural changes of carbon-based materials. Figure 3a shows the XPS survey spectra of GO and the GO–PEI–FeNPs foam composite. Compared to the XPS spectrum of GO, the GO–PEI–FeNPs foams had the characteristic N1s peak for PEI centered at ~398 eV. The high resolution C1s XPS spectrums of GO and GO–PEI–FeNPs foam composite were investigated, as shown in Figure 3b. For the GO sample, the C1s peak centered at 284.6 eV was attributed to the graphitic sp^2^ carbon atoms [39]. The C1s XPS spectra were further deconvoluted, and characteristic peaks for GO were observed at binding energies of 286.3, 287.8 and 288.9 eV corresponding to C−O, C=O and O−C=O, respectively. In the XPS spectrum of the GO–PEI foam, the C=O−C peaks at 288.8 eV decreased and were replaced by the C-N bond originating from the PEI, leading to reduction of GO upon functionalization. Moreover, addition of PEI was further confirmed with the decrease in intensity of the high resolution O1s spectrum (Figure 3c), which was shifted toward lower binding energies, possibly due to the covalent attachment of the N-group from PEI to the GO nanosheets. 

**Figure 4:**
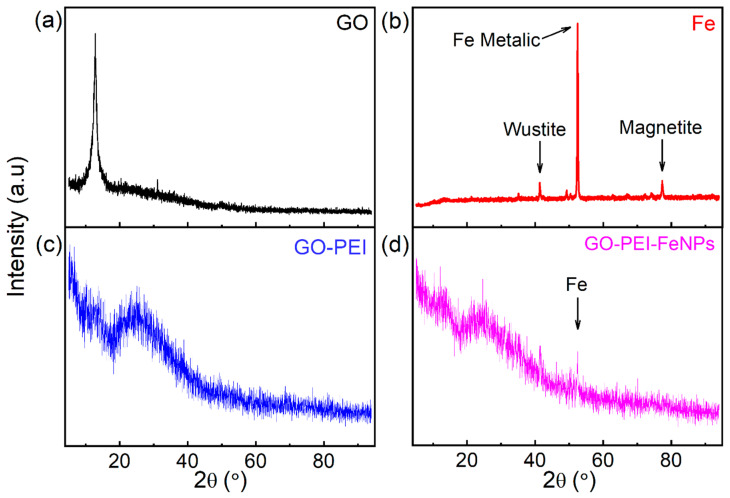
XRD diffractogram of (**a**) GO, (**b**) metallic iron, (**c**) GO–PEI and (**d**) GO–PEI–FeNPs.

Figure 4 presents the XRD pattern of GO, metallic iron, GO–PEI and GO–PEI–FeNPs. This analysis was conducted in order to visualize changes after functionalization of the GO material with the polymer and the FeNPs. In pure GO, the crystalline peak appears at 12.84° with a d-spacing (002) value of 8 Å. When the polymer is added, the peak is shifted to 23.12° with a d-spacing (002) value of 8.43 Å. This shows the behavior of a 3D assembly-reduced graphene oxide, as stipulated by other published work [38,40,41]. Comparison of the XRD pattern in Figure 4b–d shows that the peak corresponding to GO overpowered that of the FeNPs in the GO–PEI–FeNPs composite. This is expected due to the relatively small amount of nanoparticles that was added during the fabrication process. Nonetheless, it is possible to identify the most prominent XRD peak for the FeNPs at 50.58° in the foam, which could be attributed to metallic iron. This shows that during the preparation steps for the foam, the graphene oxide may have acted as a protective agent for iron nanoparticles against oxidation. 

### 3.2. Study of Adsorption of BPA, Progesterone and Norethisterone

**Figure 5:**
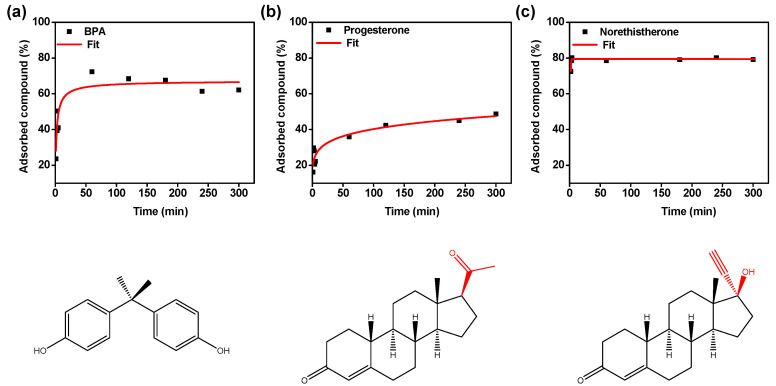
Effect of incubation time on the removal percentage of (**a**) bisphenol A (BPA), (**b**) progesterone and (**c**) norethisterone.

The adsorption times of BPA, progesterone and norethisterone were investigated by immersing the GO–PEI–FeNPs foam in aqueous solution at different time intervals. Figure 5 shows the percentage of adsorbed compound vs. time. A fast increase in adsorption is observed followed by a saturation point after a relatively short amount of time for all adsorbed compounds. Equilibrium was maintained for about 5 h with no changes for adsorption of BPA, progesterone and norethisterone. In the case of BPA (Figure 5a), the maximum % adsorbed compound was evaluated at 68% after 60 min. As for progesterone (Figure 5b), the maximum % adsorbed compound was assessed at 49% after 5 h. Finally, norethisterone (Figure 5c) had the highest % adsorbed compound of 80% after just 30 min of contact time. These different adsorption behaviors could be related to the different mechanisms controlling the adsorption process of the different endocrine disruptors. In particular, the presence of Csp^2^ bonding in all studied compounds allows a strong interaction with the GO plane via π–π stacking. Furthermore, the hydroxyl groups in BPA and the ketone and alkyne groups in the norethisterone can also be adsorbed to the GO foam via hydrogen bonding. As for progesterone, since it only possesses two ketone groups as functional groups, interactions with the foam could be weaker compared to its interactions with BPA and norethisterone, which can explain the lower adsorption by the membrane. 

**Figure 6:**
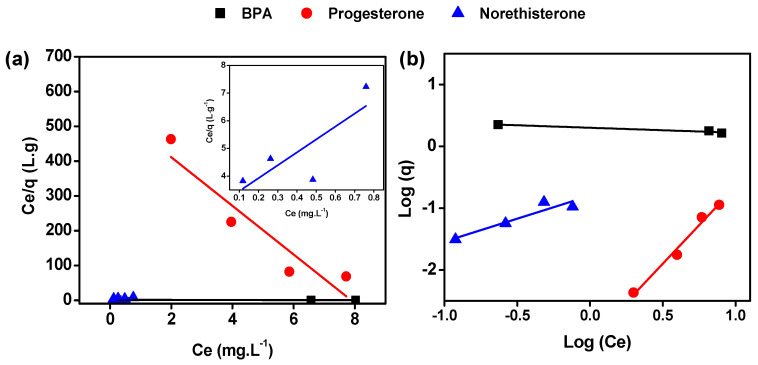
Linear regression plots of (**a**) Langmuir and (**b**) Freundlich adsorption models for BPA, progesterone and norethisterone. Inset of panel (**a**) is the Langmuir linear regression plot of norethisterone.

The adsorption capacity against the equilibrium concentration for BPA, progesterone and norethisterone at room temperature and under 60 rpm stirring rate are presented in Figure 6. Two models were investigated, the Langmuir and Freundlich models, to establish and determine the most suitable adsorption mechanisms for all compounds [32,33,42,43].

These models are described by Equations (3) and (4) and their linear equations are shown in Equations (5) and (6):

Langmuir model:

(3) $$q_{e}=\frac{q_{\max}\cdot b\cdot C_{e}}{1+b\cdot C_{e}}$$

Freundlich model:

(4) qe=K·Ce1n

where q_e_ is the adsorption capacity at equilibrium, K is the Freundlich constant related to the adsorption capacity, n is the adsorption intensity, q_max_ (mg∙g^−1^) indicates the saturated adsorption capacity, b (L∙mg^−1^) is the Langmuir constant related to the adsorption free energy and C_e_ is the concentration at equilibrium [32,33,42,43]. The equations can be linearized and the parameters q_max_, b, K and n are found by linear regression analysis:

(5) $$\frac{C_{e}}{q_{e}}=\frac{1}{b\cdot q_{\max}}+\frac{C_{e}}{q_{\max}}$$

(6) $${\log\;q}_{e}=\log\;K+\frac{1}{n}{\mathrm{logC}}_{e}$$

Table 1 summarizes different adsorption constants for linear plotting of the two models. As shown, the correlation coefficient R^2^ for BPA follows the Langmuir adsorption model. The constant n from the Freundlich model gives information on the adsorption capacity. In the case where n = 1, the adsorption is linear, but if n < 1 then the adsorption is governed by a chemical process, and finally if n > 1 then the adsorption is governed by a physical process [44]. In the case of BPA, n being inferior to 0 shows that the Freundlich model cannot be applied for adsorption of BPA by the GO membrane, which is in agreement with the literature [6]. Progesterone and norethisterone present a different adsorption process. They both have a higher linearity (R^2^) regarding the Freundlich model and different values of n. Progesterone has a n value < 1, suggesting that adsorption involves a chemical process. However, norethisterone has an n value higher than 1, indicating that this hormone exhibited favorable physisorption behavior on the GO foam. 

## 4. Conclusions

In this paper, a magnetic graphene-based foam was prepared. The composite material is stable and presents a macroporous structure which helps adsorption of large molecules with a significant removal percentage, more than 80% for some contaminants. The membrane had better affinity with BPA and norethisterone compared to progesterone due to different interactions occurring between the Csp^2^ of graphene and the H-bonding of the different remaining oxygenated groups present on the graphene surface, which agrees with previous studies [45]. The magnetic properties of the developed GO–PEI–FeNPs foam could be leveraged to easily remove the membrane from the water using a simple magnet. Therefore, the GO-based membrane is a promising adsorbent for water treatment and could be used in the future to adsorb molecules with high molecular weight. Additionally, the fabrication process of such composite materials is extremely versatile and manageable in terms of size, shape and thickness.

## Figures and Tables

**Table 1:** Isotherm parameters for adsorption of BPA, progesterone and norethisterone by the GO–PEI–FeNPs composite.

Adsorption Model/Analyte	Langmuir			Freundlich		
	b	q_max_	R^2^	n	K	R^2^
	(L∙mg^−1^)	(mg∙g^−1^)				
**BPA**	0.13	6.61	0.93	−12.43	0.5	0.92
**Progesterone**	−0.13	−0.01	0.82	0.40	1396.4	0.98
**Norethisterone**	1.56	0.21	0.49	1.35	6.3	0.83
